# The Mortality Associated with Erythema Nodosum Leprosum in Ethiopia: A Retrospective Hospital-Based Study

**DOI:** 10.1371/journal.pntd.0002690

**Published:** 2014-03-13

**Authors:** Stephen L. Walker, Eglantine Lebas, Shimelis N. Doni, Diana N. J. Lockwood, Saba M. Lambert

**Affiliations:** 1 Department of Clinical Research, Faculty of Infectious and Tropical Diseases, London, United Kingdom; 2 Centre Hospitalier Universitaire de Liège, Liege, Belgium; 3 ALERT Center, Addis Ababa, Ethiopia; University of California San Diego School of Medicine, United States of America

## Abstract

**Background:**

Erythema nodosum leprosum (ENL) is a debilitating multisystem disorder which complicates leprosy. It is characterised by fever, malaise and painful erythematous cutaneous nodules. ENL is often recurrent or chronic in nature and frequently severe. Patients often require prolonged treatment with high doses of oral corticosteroids. There are no data on the mortality associated with treated ENL.

**Methodology:**

The notes of patients who were admitted, discharged, transferred to another facility or died with a diagnosis of leprosy or a leprosy-related complication for a five year period were reviewed.

**Result/Discussion:**

414 individuals were identified from the ward database. 312 (75.4%) patient records were located and reviewed. Ninety-nine individuals had ENL and 145 had a Type 1 reaction. The median age of individuals with ENLwas 25 years. Eight patients with erythema nodosum leprosum died compared with two diagnosed with Type 1 reaction. This difference is statistically significant (p = 0.0168, Fisher's Exact Test). There is a significant mortality and morbidity associated with ENL in this Ethiopian cohort. The adverse outcomes seen are largely attributable to the chronic administration of oral corticosteroids used to control the inflammatory and debilitating symptoms of the condition.

## Author Summary

Erythema nodosum leprosum (ENL) is an immunologically mediated complication of leprosy. It is a painful debilitating multi-system condition which can occur before, during or after completion of treatment of the multi-drug therapy for leprosy. ENL is often a chronic condition requiring long-term treatment often with oral corticosteroids or thalidomide. This study shows that in Ethiopia (where thalidomide is not available) there is a significant mortality associated with ENL. It appears that much of the mortality is attributable to adverse effects of corticosteroids. This study provides additional evidence of the need for alternative agents to manage ENL particularly when thalidomide is not available, contraindicated or unaffordable.

## Introduction

Leprosy is a chronic granulomatous infection predominantly of the skin and peripheral nerves caused by *Mycobacterium leprae* [1]. 232, 857 new cases of leprosy were reported to the World Health Organization (WHO) in 2012 [2]. The treatment of the infection with multi-drug therapy (MDT) is highly effective however a significant proportion of individuals develop immune-mediated inflammatory states known as reactions.

Leprosy reactions are important because they are the major cause of nerve function impairment which leads to leprosy associated disability and its life altering consequences. Reactions may occur before, during and after successful completion of MDT. Type 1 reactions (T1R) affect patients with the borderline forms of leprosy causing inflammation in pre-existing leprosy skin lesions and neuritis [3].

Type 2 reactions or erythema nodosum leprosum (ENL) affect approximately 10% of those with borderline lepromatous (BL) leprosy and 50% of individuals with lepromatous leprosy (LL) [4]. It is acknowledged that there is a lack of good epidemiological data on the true incidence of ENL [5].

A further risk factor for developing ENL is a mean bacterial index (BI) greater than 4 on slit-skin smear [4]. It is estimated that over 50, 000 of the new leprosy patients diagnosed each year are at risk of ENL. In Ethiopia 5.3% of multibacillary patients enrolled in a field cohort study developed ENL however this cohort includes patients with borderline tuberculoid leprosy who are not at risk of ENL [6]. In Ethiopian patients with BL leprosy and LL 5% developed ENL before or during treatment with 24 months of MDT [7].

ENL is characterised by the development of crops of tender cutaneous and subcutaneous nodules in association with generalised malaise, pain and fever [1]. Other organ systems are often involved and patients may experience iritis, neuritis, rhinitis, arthritis and dactylitis, lymphadenitis, orchitis, hepatitis, peripheral oedema, and renal impairment.

The histology of ENL lesions classically shows an intense perivascular infiltrate of neutrophils throughout the dermis and subcutis [8]. Tissue oedema and vessels exhibiting fibrinoid necrosis may also be present. ENL has some features of an immune complex mediated disease. Direct immunofluorescence studies have demonstrated granular deposits of immunoglobulin and complement in the dermis in ENL lesions but not in those of uncomplicated LL disease [9]. There is evidence of T lymphocyte and macrophage activation [10].

In the majority of patients ENL is a chronic condition requiring prolonged immunosuppression [4]. Thalidomide is effective in controlling ENL and is recommended by WHO under strict medical supervision because of its severe teratogenic effects [11]. However it is not available in many leprosy endemic countries including Ethiopia and this means patients have to take large doses of oral corticosteroids often for many years. Patients often require increasing doses of prednisolone due to tachyphylaxis [12]. The clofazimine component of MDT is thought to have a protective effect with respect to ENL but this is lost once MDT is stopped. WHO recommend high dose clofazimine in conjunction with prednisolone in the management of severe ENL [11] but this requires a supply of clofazimine separate to that included in the blister packs of MDT but this is not always available (personal communication. E. Post).

A Cochrane review of the treatment of ENL highlighted the paucity of data on which to base treatment decisions and recommended well designed intervention studies [13].

ENL often affects young patients often in their 20 s and 30 s [4] and frequently restricts their ability to work and provide for their families causing financial difficulties.

Leprosy workers have long recognised that ENL is associated with a risk of death [14]. There are very few published studies examining the relationship between leprosy reactions and death. A study published in 1963 reported a significantly lower mean age at death in lepromatous patients with “lepra reactions” compared to similar patients without reactions [15]. The authors do not use the term ENL but their description is suggestive of ENL and at the time ENL was considered to be the “classical lepra reaction” [16]. Lepromatous patients with reactions had significantly increased rates of renal disease associated with persistent albuminuria compared to lepromatous patients without reactions. This was often attributed to amyloidosis [15]. The authors do not give any details about the treatments patients received for their leprosy or the “lepra reactions”. An earlier Spanish study reported a higher rate of mortality in patients with reactions compared to those without [17]. There have been no systematic studies of mortality associated with ENL since the introduction of MDT in 1982.

We wished to determine the frequency and causes of mortality associated with ENL at the ALERT Center in Addis Ababa.

## Methods

ALERT Center in Addis Ababa, Ethiopia is a referral centre for the management of patients with leprosy and other skin diseases. Patients with severe ENL are admitted for control of symptoms. Individuals with milder disease may also be admitted if there are complicating factors.

In February 2013 the database of patients admitted to the two dermatology, leprosy and HIV wards was reviewed for a 5 year period between February 2008 and January 2013. The notes of patients who were admitted, discharged, transferred to another facility or died with a diagnosis of leprosy or leprosy-related complication were retrieved and reviewed using a standard data collection tool. Data were collected on all patients diagnosed with ENL with respect to age, leprosy type, treatment, timing of presentation of ENL, number of episodes of ENL and duration of ENL. Additional details about the final admission of individuals with ENL who had died were also recorded.

An episode of ENL was defined as the occurrence of ENL requiring the institution or change of treatment (such as an increase in dosage or frequency of treatment or the addition of or switching to another drug). The nature of ENL was defined as acute for a single episode lasting less than 24 weeks. Recurrent if a patient experienced a second or subsequent episode of ENL occurring 28 days or more after stopping treatment for ENL and chronic if occurring for 24 weeks or more during which a patient has required ENL treatment either continuously or where any treatment free period has been 27 days or less.

The data were anonymised, entered in Excel and described using descriptive statistics. The Chi squared test was used to compare differences between groups.

The study (PO09/13) was approved by the AHRI/ALERT Ethics Review Committee.

## Results

414 individuals were identified from the database and the notes of 312 (75.4%) were retrieved. Ninety-nine individuals had been diagnosed with ENL, 147 with T1R, nine patients with neuritis secondary to leprosy, 11 patients had been admitted for a leprosy-related problem not due to a reaction and 46 patients were admitted for a problem other than leprosy.

The demographic and clinical features of the patients with ENL are given in Table 1. There were no significant differences between those individuals with ENL who had died and those who had not.

**Table 1:** Demographics of patients diagnosed with ENL.

Total number of patients (n = 99)		Alive n = 91 (%)	Deceased n = 8 (%)
Gender	Male	57 (62.6)	4 (50.0)
	Female	34 (37.4)	4 (50.0)
Age in years (median, [Range])	Male	28 [10–60]	25 [15–33]
	Female	24 [13–70]	22.5[19–45]
Ridley Jopling Classification	BB	1 (1)	0 (0)
	BL	14 (15.4)	2 (25)
	LL	42 (46.2)	4 (50)
	Not documented	34 (37.4)	2 (25)
Median Mean BI		3.7	3.7
HIV status	Negative	59 (64.8)	8 (100)
	Not tested	32 (35.2)	0 (0)

The timing of the occurrence of the first episode was recorded in 98 individuals. Thirty-four (34.7%) individuals presented with ENL at the time of their leprosy diagnosis, 39 (39.8%) developed ENL during treatment with MDT and 25 (25.5%) after having successfully completed a 12 month course of MDT.

ENL was acute in 19 (19.2%) individuals, recurrent in 10 (10.1%) and chronic in 70 (70.7%).

**Figure 1:**
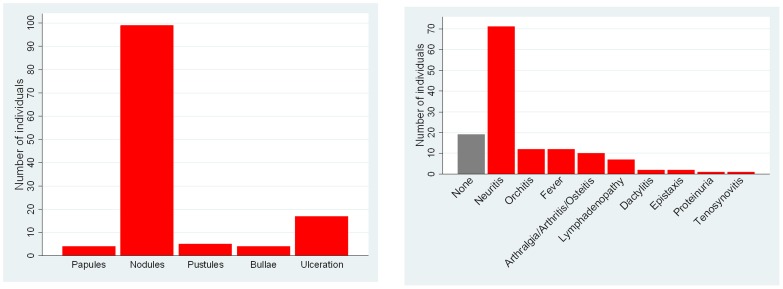
(A) Number of patients with different type of ENL skin lesions. (B) Number of patients with different extra-cutaneous manifestations of ENL.

**Figure 2:**
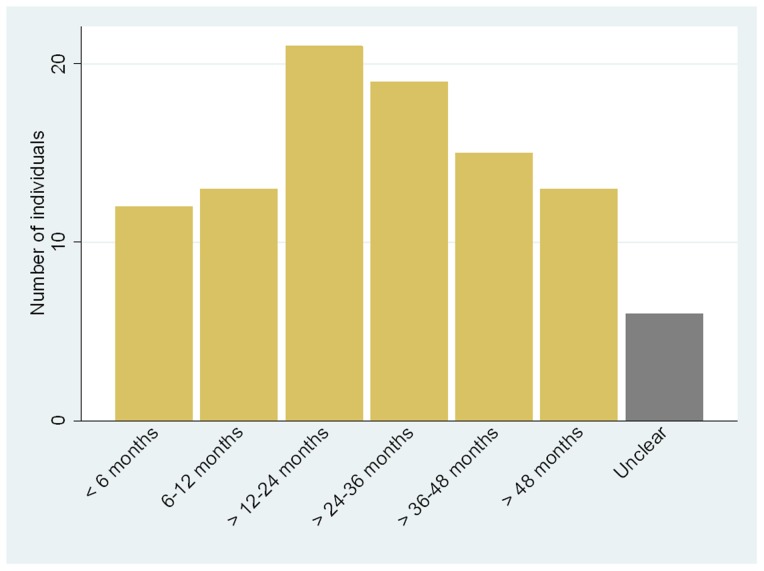
Duration of ENL.

The nature of the cutaneous lesions and other organ system involvement is shown in Fig. 1a and b. All patients had cutaneous nodules but pustular, bullous and ulcerated lesions were also seen. ENL-associated neuritis was the most frequently documented extra-cutaneous manifestation. The median number of episodes of ENL experienced was four. The duration of ENL is shown in Fig. 2.

All patients received oral prednisolone with a median starting dose of 60 mg daily. The other drugs that were used in conjunction with prednisolone at some point during the course of ENL were: clofazimine in 61 (61.2%), chloroquine in 6 (6.1%) and methotrexate and ciclosporin in one individual each.

**Figure 3:**
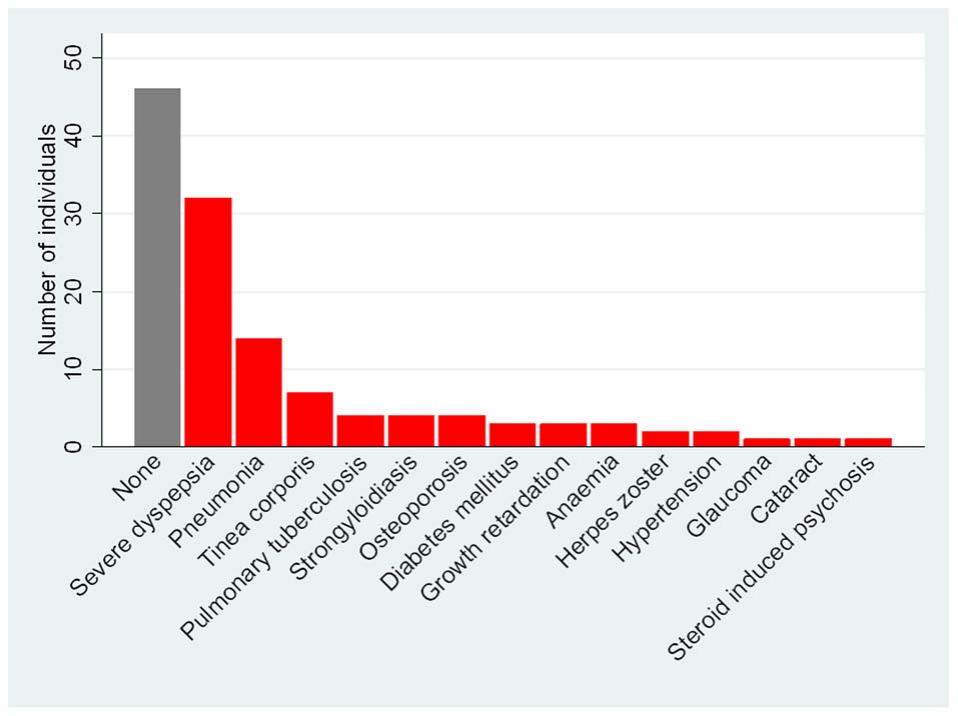
Co-morbidity associated with ENL.

**Figure 4:**
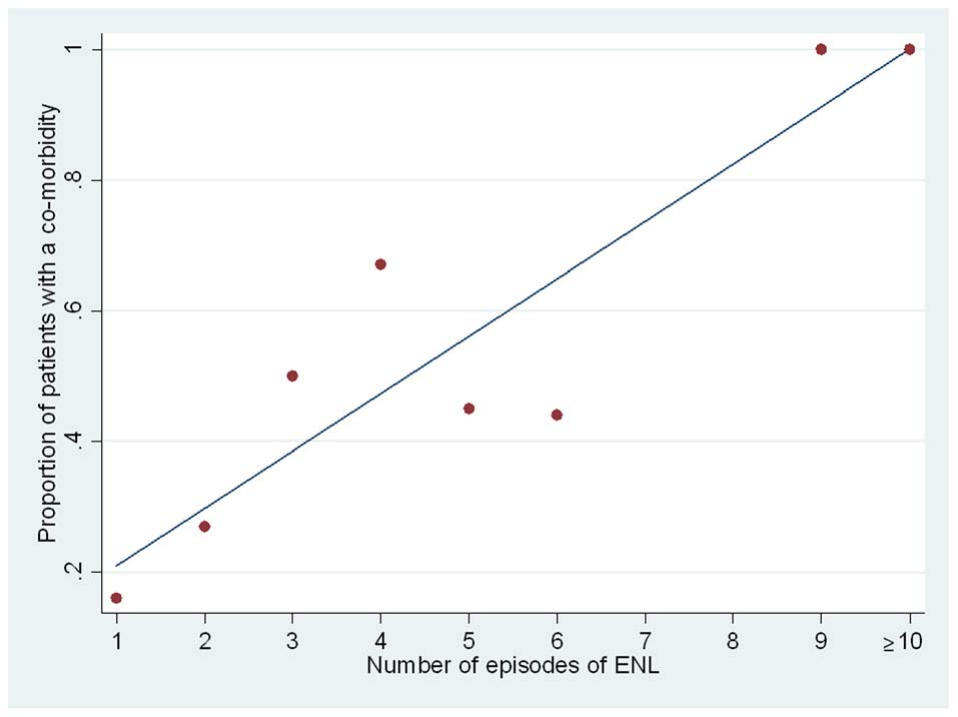
The proportion of individuals with a co-morbidity and the number of episodes of ENL.

Three individuals (3%) had a co-morbidity at the time their ENL was diagnosed. One patient had asthma, one strongyloidiasis and the third pulmonary tuberculosis. Following the diagnosis of ENL 50 (52.1%) of the remaining 96 individuals had developed aco-morbidity (Fig. 3). The co-morbidities diagnosed are shown in Fig. 4 and all of them may either be caused or exacerbated by chronic administration of high dose oral corticosteroids. There is an obvious trend in the proportion of individuals with a co-morbidity and the number of ENL episodes experienced (Fig. 4).

Two individuals (1.4%) with T1R had died compared with eight (8.1%) of those with ENL. This is statistically significant p = 0.0168, (Fisher's Exact Test). All of the patients with ENL who died were HIV negative.

Table 2. gives details of the eight individuals with ENL who died, a brief summary of their clinical course ante-mortem and the cause of death recorded in the notes by the medical staff. In four individuals it was felt that oral corticosteroid therapy was a definite contributory factor in their death and in the remaining four it was possibly contributory. In two individuals it was considered that the cause of death was possibly due to ENL itself. Seven (87.5%) of the individuals had chronic ENL which had been present for more than 18 months.

**Table 2:** Cause of mortality in ENL patients.

Gender	Age	RJ	ENL	Type and duration (months) of ENL	No. of ENL episodes	ENL features	Clinical events prior to death	Recorded cause of death	Death related to ENL	Death related to corticosteroid
Male	15	LL	Presented	Acute 4	1	Skin nodules Neuritis	Developed pulmonary tuberculosis two months after starting prednisolone	Diabetic ketoacidosis	No	Definite
Female	19	----	Presented	Chronic >36	>10	Skin nodules Fever Epistaxis	Thrombocytopenia secondary to sepsis with severe epistaxis	Not recorded	Possible	Possible
Female	20	LL	During MDT	Chronic >24	>10	Skin nodules Neuritis	Admitted with septic shock while taking prednisolone 60 mg daily	Septic shock	No	Definite
Male	22	BL	During MDT	Chronic 59	>10	Skin nodules Neuritis	Acute hepatitis B while taking prednisolone	Viral hepatitis leading to multiorgan failure	No	Possible
Female	25	LL	Presented	Chronic >18	4	Skin nodules Neuritis	Developed cough while taking prednisolone 55 mg daily. Deteriorated with fever, dyspnoea and died	Pneumonia secondary to immunosuppre-ssion	No	Definite
Male	28	LL	Presented	Chronic >36	>10	Skin nodules with ulceration Neuritis Orchitis	Epigastric pain, anaemia and hepatosplenomegaly.	Multiorgan failure	Possible	Possible
Male	33	---	During MDT	Chronic 36	4	Skin nodules Neuritis Orchitis	Admitted with weight loss, night sweats, cough and pleurisy while taking prednisolone 40 mg daily (self-medicating). AFB in sputum. Died 4 weeks later.	Not recorded	No	Definite
Female	45	BL	During MDT	Chronic >24	4	Skin nodules Fever	Developed herpes zoster. Collapsed with cardio-respiratory arrest. History of pre-existing asthma.	Not recorded	No	Possible

## Discussion

The data from this retrospective study must be interpreted with caution although almost 75% of case notes were available. The data extracted were reliant on the findings recorded by the clinicians at the time the patients were seen. The information about deaths is also reliant on the clinical records as no post-mortem autopsies were performed.

This is the first study to report that a significant proportion of Ethiopian patients with ENL are dying and that their deaths appear largely attributable to prolonged treatment with oral corticosteroids. The individuals who succumbed were young and none of the deaths were related to HIV infection. We believe that our data underestimates the mortality associated with ENL and its treatment. The lack of any data on the mortality associated with ENL (apart from occasional case reports) [18] since the publication by Brusco and Masanti [15] may be due to better prognosis due to MDT and the use of drugs such as thalidomide and clofazimine. However we believe it is more likely that patients who die are likely to be considered as simply “lost to follow up” or there may be reporting bias due to a reticence on behalf of health workers to report such negative outcomes in patients with ENL. This may give a falsely reassuring picture of ENL. Mortality data from other centres where ENL patients are treated would be useful in further assessing the impact of ENL and understanding the factors that result in patient deaths.

The significant difference in the number of deaths in those with ENL compared to individuals with T1R is likely due to the shorter duration of T1R which are usually treated with reducing doses of oral prednisolone over the course of six months [3]. Many patients with T1R also require additional corticosteroids but not for as long as patients with ENL [19]. The adverse effects of prednisolone were examined in the TRIPOD studies which recruited 815 participants and were conducted in Bangladesh and Nepal. These three randomised, double-blind studies examined the role of 16 weeks of prednisolone as: prophylaxis for reactions and neuritis, in the treatment of mild sensory impairment and, in nerve function impairment present for more than six months [20], [21], [22]. There were no significant differences in major adverse events between the prednisolone treated and placebo groups [23].

The ENL seen in this cohort is typical of that described by other authors in terms of clinical features, number of episodes and duration [19], [24], [25]. Neuritis was the most frequent non-cutaneous manifestation. It is notable that 12 (19.7%) men were diagnosed as having orchitis and that fever which is commonly regarded as one of the hallmarks of ENL was only recorded in 12.1% of individuals.

Forty-seven (50.5%) of the 93 patients for whom it was possible to calculate the duration of ENL experienced chronic disease lasting more than 24 months and 13 (14%) had ENL for more than 4 years. This is not surprising given that ALERT Center is a referral hospital and that the methodology of this study relied on a database of patients who had been admitted. However this feature of ENL is described in other cohorts and demonstrates that ENL poses a significant and disproportionate burden on health services. Of the 19 patients who were diagnosed as having acute ENL it is likely that a sizeable proportion may go on to have further episodes and thus become either recurrent or chronic cases as a similar study from India showed that individuals with acute ENL made up only 8% of all ENL cases [4].

The chronic nature of ENL means that patients require long term treatment. There is longstanding experience of the effectiveness of thalidomide in controlling ENL. However there are few alternatives to long term treatment with oral corticosteroids for patients living in places where thalidomide is not available, for those in whom thalidomide is contraindicated or its use is limited by adverse effects and, for those who do not wish to take the drug or cannot afford it. This leads to unacceptably high rates of adverse effects as seen in this cohort in which those patients experiencing more episodes of ENL are more likely to experience severe co-morbidities attributable to corticosteroids. Milder adverse effects of corticosteroids are likely to be underestimated by this study as they are less likely to have been recorded in the patients' case notes. The long term implications for patients with ENL treated with high dose corticostreroids are unclear. Sugumaran reported high rates of adverse effects due to corticosteroids in 249 patients with ENL [26]. He stressed the need to identify agents other than corticosteroids which would be useful in the management of ENL and this sentiment was reiterated by the authors of the Cochrane review [13] and by the participants of an international workshop on ENL [27]. Other drugs may play a role as corticosteroid-sparing agents in ENL or as true alternatives to corticosteroids but it is vital that evidence is gathered to assess their efficacy and safety. There also needs to be wider public debate about the role of thalidomide and how it might be used safely in the management of ENL in those countries where it is not currently available.

It is essential that robust evidence-based local guidelines are produced to facilitate the management of leprosy patients with ENL in order to try and minimise adverse outcomes including premature deaths. These guidelines may include the early use of corticosteroid sparing agents and there will need to be an adequate, reliable and affordable supply of such drugs.
